# Identification of Factors Related to the Quality of Lymphadenectomy for Lung Cancer: Secondary Analysis of Prospective Randomized Trial Data

**DOI:** 10.3390/jcm12113780

**Published:** 2023-05-31

**Authors:** Piotr Gabryel, Magdalena Roszak, Piotr Skrzypczak, Anna Gabryel, Dominika Zielińska, Magdalena Sielewicz, Alessio Campisi, Mariusz Kasprzyk, Cezary Piwkowski

**Affiliations:** 1 Department of Thoracic Surgery, Poznan University of Medical Sciences, Szamarzewskiego 62 Street, 60-569 Poznan, Polandcpiwkow@ump.edu.pl (C.P.); 2 Department of Computer Science and Statistics, Poznan University of Medical Sciences, Rokietnicka 7 Street, 60-806 Poznan, Poland; 3 Department of Thoracic Surgery, University and Hospital Trust–Ospedale Borgo Trento, Piazzale Aristide Stefani 1, 37126 Verona, Italy

**Keywords:** lung cancer, surgery, lobe-specific, lymphadenectomy, minimally invasive surgery, lobectomy, thoracoscopy/VATS, LigaSure, energy device

## Abstract

The outcomes of non-small cell lung cancer surgery are influenced by the quality of lymphadenectomy. This study aimed to evaluate the impact of different energy devices on lymphadenectomy quality and identify additional influencing factors. This secondary analysis of the prospective randomized trial data (clinicaltrials.gov: NCT03125798) compared patients who underwent thoracoscopic lobectomy with the LigaSure device (study group, *n* = 96) and monopolar device (control group, *n* = 94). The primary endpoint was the lobe-specific mediastinal lymphadenectomy. Lobe-specific mediastinal lymphadenectomy criteria were met in 60.4% and 38.3% of patients in the study and control groups, respectively (*p =* 0.002). In addition, in the study group, the median number of mediastinal lymph node stations removed was higher (4 vs. 3, *p =* 0.017), and complete resection was more often achieved (91.7% vs. 80.9%, *p =* 0.030). Logistic regression analysis indicated that lymphadenectomy quality was positively associated with the use of the LigaSure device (OR, 2.729; 95% CI, 1.446 to 5.152; *p =* 0.002) and female sex (OR, 2.012; 95% CI, 1.058 to 3.829; *p =* 0.033), but negatively associated with a higher Charlson Comorbidity Index (OR, 0.781; 95% CI, 0.620 to 0.986; *p =* 0.037), left lower lobectomy (OR, 0.263; 95% CI, 0.096 to 0.726; *p =* 0.010) and middle lobectomy (OR, 0.136; 95% CI, 0.031 to 0.606, *p =* 0.009). This study found that using the LigaSure device can improve the quality of lymphadenectomy in lung cancer patients and also identified other factors that affect the quality of lymphadenectomy. These findings contribute to improving lung cancer surgical treatment outcomes and provide valuable insights for clinical practice.

## 1. Introduction

According to the World Health Organization data, lung cancer is one of the most prevalent cancers worldwide and the leading cause of cancer death [1]. The most common type of lung cancer, diagnosed in approximately 85% of patients, is non-small cell lung cancer (NSCLC) [2]. The cancer stage, according to the TNM classification, determines the prognosis and treatment method [3]. The standard of care for early-stage NSCLC is anatomical lung resection and lymphadenectomy [3]. Long-term survival largely depends on the TNM stage and the completeness of surgery, but the quality of lymphadenectomy also affects the outcomes [4,5]. Several guidelines have been issued to standardize lymphadenectomy and ensure its quality. These guidelines specify the lymph node stations that should be removed during lung cancer surgery but differ in the recommended extent of the lymphadenectomy. For example, the National Comprehensive Cancer Network (NCCN) indicates that N1 and N2 node resection and mapping should be routine components of lung cancer surgery, with a minimum of 3 N2 stations sampled or dissected [6]. European Society of Thoracic Surgeons guidelines suggest the removal of virtually all mediastinal lymph nodes on the operated side [7]. The International Association for the Study of Lung Cancer (IASLC) guidelines recommend either the systematic nodal dissection (SMLND) with the complete excision of the mediastinal fat and enclosed lymph nodes or the lobe-specific systematic nodal dissection (L-SMLND), in which lymph nodes in the area of lymph drainage from a specific lobe of the lung are removed (Appendix A) [3]. Although these guidelines are widely used and allow for the standardization of procedures, they also have disadvantages. First, several studies have demonstrated that the greater the number of lymph nodes removed during surgery, the better the long-term outcome of lung cancer treatment [8,9]. However, the number of lymph nodes is not included in the guidelines mentioned above. Secondly, the guidelines do not have recommendations regarding the analysis and the control of factors that could affect the quality of lymphadenectomy. Certain factors related to the quality of the lymph node dissection, such as age, side of surgery and extent of resection [10,11], are not modifiable. On the other hand, certain technical innovations can improve the quality of lymphadenectomy. For example, using a lymph node collection kit during lung cancer surgery has been shown to enhance pathologic nodal staging quality significantly [12]. Moreover, a recent prospective randomized trial indicated that the energy device used for dissection could impact the quality of mediastinal lymphadenectomy. However, the detailed analysis of those issues was beyond the scope of that trial [13].

Despite the evidence supporting the importance of accurate lymphadenectomy, several studies demonstrated that compliance with the guidelines and the number of lymph nodes removed is often low [12,14]. For this reason, the identification of factors that influence the quality of lymphadenectomy and the assessment of the role of the type of electrosurgical device used for dissection may be of value.

The study aimed to determine whether the type of the electrosurgical device (monopolar or advanced bipolar device) was related to the quality of mediastinal lymphadenectomy and to identify other factors that could influence the accuracy of lymph nodes dissection in patients undergoing video-assisted thoracoscopic surgery (VATS) lobectomy for non-small cell lung cancer (NSCLC).

## 2. Materials and Methods

The Bioethics Committee of the Poznan University of Medical Sciences waived the need for ethics approval and the need to obtain consent for secondary analysis and publication of the anonymized data of the prospective randomized trial (approval of the Bioethics Committee of the Poznan University of Medical Sciences, Poznan, Poland, 16 June 2016, number 764/16; registered at www.clinicaltrials.gov identifier: NCT03125798), supplemented with detailed histopathological data on nodal dissection.

The study included patients who underwent VATS lobectomy with lymphadenectomy for NSCLC at the Department of Thoracic Surgery of Poznan University of Medical Sciences between 15 May 2018 and 17 June 2021. Exclusion criteria were neoadjuvant chemo- and/or radiotherapy and any prior surgical procedure involving the mediastinum, lung, or chest wall. In addition, to conduct an analysis corresponding to the objectives of the present research, compared to the original prospective randomized study [13], we also excluded patients with conversion to thoracotomy and non-malignant or metastatic histology.

Before the surgery, all patients underwent a chest computed tomography scan, abdominal ultrasound, electrocardiography, pulmonary function tests, diffusing capacity for carbon monoxide, positron emission tomography/computed tomography and fiberoptic bronchoscopy. If indicated, echocardiography, exercise testing and invasive mediastinal staging were done. The perioperative risk was assessed according to the American Society of Anesthesiologists scale and the Thoracic Revised Cardiac Risk Index (ThRCRI). In addition, Charlson Comorbidity Index (CCI) was calculated to assess the burden of comorbidities.

Patients who met the inclusion criteria and gave written informed consent to participate in the original study [13] were assigned to either the study group (tissue dissection with the LigaSure device) or the control group (dissection with monopolar device) using a simple randomization technique with a 1:1 ratio. The assignment sequence was created with a random number generator (www.graphpad.com, accessed on 9 April 2018). To conceal assignments, we used opaque, sealed envelopes prepared before the study and opened just before the surgery. From this point, patients’ assignments were known to the operating surgeon. Patients, nurses, and doctors engaged in the patient’s perioperative care, including chest tube management and removal, were blinded to the type of intervention [13].

Surgery was performed under general anesthesia by one of the five board-certified thoracic surgeons experienced in thoracoscopic anatomical resections. The VATS approach consisted of a 4–5-cm-long utility incision and usually the placement of one or two thoracic trocars. No rib spreader was used. For the dissection of tissues and lymphadenectomy, either a monopolar electrosurgical device or the LigaSure (Medtronic, Dublin, Ireland) device was used. Endostaplers were used to divide the pulmonary vessels, bronchi, and interlobar fissures. Resected lobe was removed from the pleural cavity in a plastic bag. Lymphadenectomy was performed during tissue dissection during surgery and was completed after lobectomy. At the end of the surgery, one 24-F chest tube was inserted into the pleural cavity and connected to a digital drainage system (Thopaz+; Medela, Baar, Switzerland). The chest tube was removed after the resolution of the air leak and when the fluid volume was <250 mL for 24 h.

We documented each group’s preoperative, surgical, postoperative, and histopathological characteristics. Data for the present study were obtained from the Case Report Forms from the original randomized study [13] and supplemented with detailed histopathological data, including data on the lymph node stations dissected, the number of lymph nodes removed from each nodal station, lymph node metastases, invasion of the nodal capsule, metastases in the highest nodal station dissected and the completeness of the resection. Clinical outcomes and histological type were classified in accordance with the European Society of Thoracic Surgery/Society of Thoracic Surgeons definitions [15] and the World Health Organization classification [16], respectively, and the TNM stage, lobe-specific lymph node dissection and completeness of resection in accordance with the IASLC guidelines (Appendix A) [3].

The primary endpoint was the lobe-specific mediastinal lymph node dissection (L-SMLND), defined as the dissection of mediastinal N2 lymph node stations according to the IASLC guidelines (Appendix A) [3,5], assessed in the postoperative histopathological examination. We decided on this primary endpoint because it depended solely on the quality of the lymphadenectomy performed by the surgeon, and it was not influenced by the quality of lung dissection during the histopathological examination. The L-SMLND included stations 2R, 4R and 7 for right upper lobectomy and right middle lobectomy, stations 4R, 7, and 8 or 9 for right lower lobectomy, stations 5, 6 and 7 for left upper lobectomy and stations 7, 8 and 9 for left lower lobectomy. Secondary endpoints were the number of lymph node stations dissected, the number of lymph nodes removed, and the completeness of resection.

### Statistical Analysis

The analyzed data were expressed as mean ± standard deviation, median, minimum, maximum values, interquartile range (Q1 lower quartile, Q3 upper quartile), or percentage, as appropriate. The relationship between variables was analyzed using Spearman’s rank correlation coefficient. The normality of distribution was tested using the Shapiro–Wilk test and equality of variances was checked using Levene’s test. A comparison of two unpaired groups was performed using the unpaired *t*-test for data that followed a normal distribution and had homogeneity of variances or the Mann–Whitney U-test. Categorical data were analyzed using the χ^2^ test when the sample size was larger than 40 and all expected values were greater than ten; for other situations, the exact test of Fisher or χ^2^ test with Yate’s correction was used. All results were considered significant at *p* < 0.05. Factors that obtained *p* < 0.05 in univariate analysis were then analyzed in logistic regression. The sample size for the original study was calculated to assess whether the LigaSure device was superior to the monopolar device in terms of the postoperative chest drainage volume [13]. Post-hoc power calculations were performed for the primary and secondary endpoints to evaluate the statistical power of the present study. Data manipulation and all calculations were performed in IBM^®^ SPSS^®^ Statistics version 27th (PS Imago Pro 8).

## 3. Results

The study included 190 patients after VATS lobectomy for NSCLC, assigned to either the study group (LigaSure device, *n* = 96) or the control group (monopolar device, *n* = 94). The groups did not differ regarding the baseline, surgical and postoperative, and general histological characteristics listed in Table 1, Table 2 and Table 3, respectively.

We found that the lobe-specific lymphadenectomy guidelines for N2 nodal stations were met in 60.4% and 38.3% of patients in the study and control groups, respectively; these differences were significant (*p* = 0.002). The guidelines for all (N2 and N1) stations were met in 40.6% of patients in the study group and 16% of patients in the control group (*p* < 0.001). Compliance with the NCCN guidelines did not differ between the groups (Table 4).

The median numbers of N2 lymph nodes removed were higher in the study group compared to the control group, but the difference was not significant (11 [IQR, 7–15] vs. 9 [IQR, 6–13], *p* = 0.080). The median numbers of N1 lymph nodes removed did not differ between the groups (Table 4).

**Figure 1:**
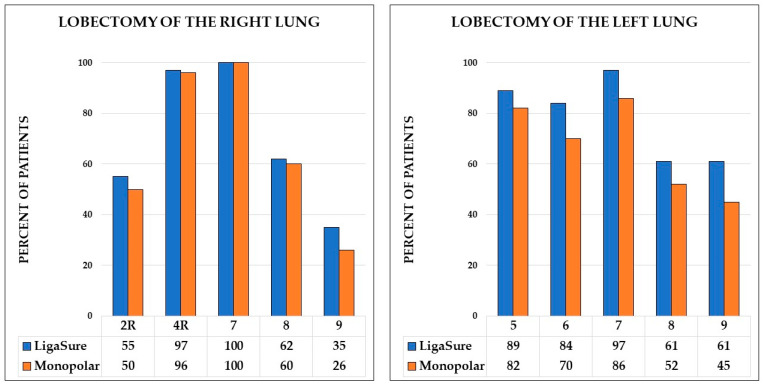
Percentage of patients with removed lymph nodes stations for right and left lobectomies.

The median numbers of N2 lymph node stations dissected were higher in the study group compared to the control group (4 [IQR, 3–4] vs. 3 [IQR, 3–4], *p =* 0.017). However, the median numbers of N1 lymph node stations did not differ between the groups (Table 4). Detailed data on the lymph node stations dissected are presented in Figure 1.

Surgery was complete (R0) in 91.7% and 80.9% of patients in the study and control groups, respectively (*p =* 0.030). In the remaining patients, according to the IASLC guidelines, the surgery was either incomplete or completeness was uncertain (Table 3).

Post-hoc power for N2 L-SMLND, number of lymph node stations dissected, and completeness of resection was 86.7%, 70% and 58.3%, respectively.

The univariable analysis found five variables associated with compliance to the lobe-specific mediastinal lymphadenectomy guidelines, as follows: LigaSure device (*p =* 0.002), type of lobectomy (*p =* 0.018), female sex (*p =* 0.045), ThRCRI (*p =* 0.041) and CCI (*p =* 0.009) (Table 5). In addition, as there was a significant, moderately strong correlation between CCI and ThRCRI (*p =* 0.001, Spearman’s rho = 0.471), the data analyzed by logistic regression were: type of device, type of lobectomy, sex and CCI.

Logistic regression analysis indicated that lymphadenectomy quality was positively associated with the use of the LigaSure device (OR, 2.729; 95% CI, 1.446 to 5.152; *p =* 0.002) and female sex (OR, 2.012; 95% CI, 1.058 to 3.829; *p =* 0.033), but negatively associated with a higher Charlson Comorbidity Index (OR, 0.781; 95% CI, 0.620 to 0.986; *p =* 0.037), left lower lobectomy (OR, 0.263; 95% CI, 0.096 to 0.726; *p =* 0.010) and middle lobectomy (OR, 0.136; 95% CI, 0.031 to 0.606, *p =* 0.009).

## 4. Discussion

The study showed that using the LigaSure device was related to better compliance with IASLC lymphadenectomy guidelines, higher numbers of dissected N2 nodal stations and higher rates of complete resections. We also found that mediastinal lymph node dissection quality was better in females and worse in patients with higher Charlson Comorbidity Index and after left lower and right middle lobectomy.

High-quality lymphadenectomy is essential for NSCLC surgical treatment because it improves staging accuracy, guides adjuvant treatment, and affects long-term treatment outcomes [17]. However, the relationship between the quality of lymphadenectomy and the type of electrosurgical device used is not well understood. A prospective randomized trial found that more lymph nodes were removed in patients with the LigaSure device [13]. In that trial, lymph nodes were counted at the end of the surgery by another surgeon, who was blinded to the patient’s group assignment. In addition, in some patients, the number of lymph nodes could have also included some of the lymph nodes from groups 10 and 11, which made the results more challenging to analyze and interpret. In the current study, we analyzed lymph nodes in groups N2 and N1 separately, based on the results of histopathological examination. This approach allowed for a more accurate assessment of the impact of the electrosurgical device on mediastinal lymphadenectomy and eliminated the risk of observer bias.

The higher lymphadenectomy quality was most likely related to the improved design of the LigaSure device. The insulated tips of the device allow for higher energy generation and effective and safe tissue fusion [18]. As pointed out by Nakazawa et al., the goal of lymphadenectomy should be to remove en-bloc all tissues containing lymph nodes in 3-dimensional spaces limited by adjacent organs [19]. Such lymphadenectomy requires dissection directly at the esophagus, bronchi, and vessels. Energy dissipation from the non-insulated tips of the monopolar electrosurgical devices creates a risk of organ damage, may necessitate dissection with a greater tissue margin, and could result in less precise lymphadenectomy [20]. Lower energy dissipation at the LigaSure tips reduces the risk of thermal injury, allows tissue dissection directly adjacent to key organs and facilitates lymphadenectomy [21]. This may be particularly important for deep lymph nodes between other anatomical structures, such as left paratracheal and subcarinal nodes [22].

The study also demonstrated that, in addition to the type of energy device, the quality of lymphadenectomy could be affected by other factors, such as gender, comorbidities, and the kind of lobectomy.

The reason for better lymphadenectomy in females is unclear. It may be related to gender-based differences in baseline characteristics, cancer type, stage and location, or other unforeseen factors [23,24]. Studies on the relation of sex-based differences to surgical lung cancer treatment results showed that long-term survival was better in females [25]. This finding was associated with a lower incidence of postoperative complications and earlier stages of NSCLC in females [26]. More thorough lymphadenectomy, more accurate staging and better qualification for adjuvant treatment could be another reason for improved overall survival in females.

Patients operated on for lung cancer often suffer from other tobacco-related diseases [27]. A significant burden of comorbidities results in higher complication rates and worse long-term outcomes and may be an indication to limit the extent of the lung parenchyma resection [28,29]. No guidelines or studies recommend limiting the extent of lymphadenectomy in patients with severe comorbidities. However, less thorough lymphadenectomy in patients with multiple, severe comorbidities demonstrated by the present study may result from efforts to minimize the surgical injury, shorten the duration of surgical operation, and limit the risk of postoperative complications. This is most likely not a favorable situation, as it leads to less accurate lung cancer staging and may impair the qualification for the adjuvant treatment. This could adversely affect the outcomes of treatments and requires further research.

Univariate analysis showed that lymphadenectomy quality is related to Charlson Comorbidity Index (CCI) and the Thoracic Revised Cardiac Risk Index (ThRCRI). Both indicators reflect the patient’s burden of comorbidities, and we found a strong correlation between them. For this reason, we included only CCI in the multivariate analysis. However, the relationship between lymph node dissection and comorbidities discussed above most likely applies to ThRCRI.

Less accurate lymphadenectomy for left lower lobectomy resulted mainly from differences in IASLC lymphadenectomy criteria for left lower and right lower lobectomy [5]. To fulfil the IASCL guidelines for L-SMLND for right lower lobectomy, station 8 or 9 nodes should be removed. The guidelines for the left lower lobe are more rigorous and challenging to follow. In this case, both station 8 and 9 nodes should be resected. We assume that the differences in the L-SMLND criteria were the main cause of the differences in the lymphadenectomy quality between the lobes.

An interesting finding was worse lymphadenectomy in patients with the middle lobectomy. In this group, more patients had T1 and T2 tumors compared to the entire study group. These results are consistent with studies by Pawelczyk et al. [30] and Edwards et al. [31], who demonstrated that lymphadenectomy was less thorough in patients operated for smaller tumors. Moreover, the study by Mazza et al. revealed that compared to other types of lobectomy, patients with pulmonary middle lobectomy had smaller tumors, less accurate lymphadenectomy and lower 5-year survival [32]. The reason for the inferior quality of lymphadenectomy for the middle lobectomy is unclear. It may result from more difficult access to mediastinal lymph nodes, especially in the subcarinal region. In addition, the perception of the middle lobectomy as the less extensive procedure for less advanced tumors, compared to the other lobectomies, could lead to less extensive lymphadenectomy. Regardless of the cause, less thorough lymphadenectomy may be associated with poorer long-term outcomes.

The last finding of the study was the higher rate of complete (R0) resections in patients with lymph node dissection with the LigaSure device. Upon closer analysis, we found that the rates of incomplete resection (R1) were similar between the two groups. The differences resulted primarily from the higher frequency of uncertain resections (Run) in the group of patients operated on with a monopolar device. In all cases, the direct reason for uncertain resection was involved (metastatic) highest mediastinal lymph node. It can be assumed that if lymphadenectomy were more thorough and involved more nodal groups, the incidence of metastases in the highest removed lymph nodes would be lower, and the frequency of radical resection would be higher.

High-energy devices, such as the LigaSure device, also have some limitations. First, the cost of purchasing a generator and disposable devices is high, limiting its use. Secondly, although the use of the LigaSure device is safe, some thermal spread may still occur, and standard safety precautions must be followed to avoid damaging adjacent structures [20,21].

The main limitation was the relatively small number of subjects. Although the sample size was sufficient to analyze the primary and secondary endpoints, it prevented the analysis of other factors that could have been related to the quality of the lymphadenectomy, such as the clinical T stage and the operating surgeon. The study was also underpowered to analyze differences between the devices within individual nodal stations. Another limitation was that the study was conducted in a single center, which may reduce generalizability to broader clinical practice. Finally, unblinded design—it was not possible to conceal assignment from the surgeons that conducted operations—might have introduced treatment bias; however, observer bias regarding the primary and secondary endpoints was unlikely, as those were assessed in the histopathological examination.

## 5. Conclusions

The study demonstrated that using the LigaSure device improves the quality of lymphadenectomy in patients undergoing minimally invasive lobectomy for lung cancer. This is an important finding because it may enhance the accuracy of cancer staging, facilitate adjuvant treatment planning, and possibly improve treatment outcomes. However, high-energy bipolar devices are expensive, and the advantages demonstrated by the previous studies, such as lower volume and shorter duration of chest drainage, have not fully justified their purchase. Nevertheless, the higher quality of lymphadenectomy is an important advantage, supporting the widespread use of high-energy devices for lung cancer surgery.

The study also found other factors, such as sex, burden of comorbidities and type of lobectomy, that may affect the quality of lymphadenectomy. In the groups of male patients with higher Charlson Comorbidity Index, undergoing right middle and left lower lobectomy, special attention should be paid to the appropriate accuracy of lymph node dissection. Strict adherence to lymphadenectomy guidelines may improve the completeness of surgery and the long-term outcomes of lung cancer treatment.

Lymphadenectomy is an important part of the surgical treatment of lung cancer, but the factors that may affect it are poorly studied. Further multicenter studies, including larger patient cohorts, are needed to evaluate the role of the high-energy devices, identify other factors, and develop models that predict the quality of lymph node dissection.

## Figures and Tables

**Table 1:** Comparison of baseline characteristics between LigaSure and Monopolar groups.

Variables	LigaSure *n* = 96	Monopolar *n* = 94	*p* Value
Age, years, mean (SD)	66.3 (SD: 7.3)	66.6 (SD: 7.1)	0.712
Sex, *n* (%)			0.864
Male	55 (57.3)	55 (58.5)	
Female	41 (42.7)	39 (41.5)	
Body Mass Index, kg/m^2^, mean (SD)	26.5 (SD: 4.2)	26.9 (SD: 5.3)	0.761
ppFEV_1_%, mean (SD)	63 (SD: 16)	64 (SD: 17)	0.912
ppDLCO%, mean (SD)	70 (SD: 25)	73 (SD: 21)	0.402
Comorbidities, *n* (%)	77 (80.2)	83 (88.3)	0.126
Chronic obstructive pulmonary disease	32 (33.3)	32 (34.0)	0.917
Coronary arterial disease	14 (14.6)	18 (19.1)	0.400
Cerebrovascular disease	6 (6.2)	4 (4.3)	0.771
Peripheral arterial disease	12 (12.5)	12 (12.8)	0.956
Hypertension	49 (51.0)	53 (56.4)	0.460
Diabetes mellitus	12 (12.5)	28 (29.8)	0.003 *
Chronic kidney disease	2 (2.1)	2 (2.1)	0.628
Other	29 (30.2)	28 (29.8)	0.949
Thoracic Revised Cardiac Risk Index, *n* (%)			0.101
Class A	79 (82.3)	73 (77.7)	
Class B	14 (14.6)	21 (22.3)	
Class C	3 (3.1)	0 (0)	
Charlson Comorbidity Index, median (IQR)	3 (IQR, 2 to 4)	3 (IQR, 2 to 5)	0.209

ppFEV_1_% = predicted postoperative percentage of calculated forced expiratory volume in 1 s; ppDLCO% = predicted postoperative percentage of calculated diffusion lung capacity for carbon monoxide; SD = standard deviation; IQR = interquartile range. * Statistically significant (*p* < 0.05).

**Table 2:** Comparison of surgical and postoperative characteristics between LigaSure and Monopolar groups.

Variables	LigaSure *n* = 96	Monopolar *n* = 94	*p* Value
Type of thoracoscopic approach, *n* (%)			0.408
Multiportal	90 (93.8)	86 (91.5)	
Uniportal	6 (6.2)	8 (8.5)	
Side, *n* (%)			0.315
Left	38 (39.6)	44 (46.8)	
Right	58 (60.4)	50 (53.2)	
Type of lobectomy, *n* (%)			0.507
Right upper	39 (40.6)	31 (33.0)	
Right middle	8 (8.3)	4 (4.3)	
Right lower	11 (11.5)	15 (16.0)	
Left upper	24 (25.0)	27 (28.7)	
Left lower	14 (14.6)	17 (18.1)	
Duration of surgery, min, mean (SD)	117 (SD: 35)	120 (SD: 40)	0.607
Estimated blood loss, mL, median (IQR)	100 (IQR, 50 to 125)	100 (IQR, 50 to 120)	0.774
Complications, *n* (%)	24 (25.0)	25 (26.6)	0.801
Prolonged air leak	9 (9.4)	10 (10.6)	0.771
Residual air space	8 (8.3)	7 (7.5)	0.820
Atrial fibrillation	6 (6.3)	3 (3.2)	0.515
Chylothorax	1 (1.0)	2 (2.1)	0.985
Pulmonary complications	4 (4.2)	1 (1.1)	0.286
Other	2 (2.1)	5 (5.3)	0.424
Chest tube duration, days, median (IQR)	2 (IQR, 2 to 4)	3 (IQR, 2 to 4)	0.417
Hospital stay, days, median (IQR)	5 (IQR, 4 to 7)	5 (IQR, 4 to 7)	0.325
Readmission, *n* (%)	1 (1.0)	0	0.991

SD = standard deviation; IQR = interquartile range.

**Table 3:** Comparison of histopathological characteristics between LigaSure and Monopolar groups.

Variables	LigaSure *n* = 96	Monopolar *n* = 94	*p* Value
Histology, *n* (%)			0.618
Adenocarcinoma	53 (55.2)	51 (54.3)	
Squamous cell carcinoma	33 (35.4)	28 (29.8)	
Other carcinoma	10 (9.4)	15 (15.9)	
Completeness of resection, *n* (%)			0.030 *
Complete resection (R0)	88 (91.7)	76 (80.9)	
Incomplete or uncertain resection (R1/Run)	8 (8.3)	18 (19.1)	
Tumor invasion of resection margins	2 (2.1)	3 (3.2)	
Extracapsular involvement of lymph nodes	2 (2.1)	3 (3.2)	
Involved highest mediastinal lymph node	4 (4.2)	12 (12.8)	
Pathological stage, *n* (%)			0.215
Stage I	53 (55.2)	50 (53.2)	
Stage II	31 (32.3)	25 (26.6)	
Stage III	12 (12.5)	19 (20.2)	

* Statistically significant (*p* < 0.05).

**Table 4:** Comparison of lymphadenectomy, clinical and pathological nodal staging, and nodal upstaging between the LigaSure and the Monopolar groups.

Variables	LigaSure *n* = 96	Monopolar *n* = 94	*p* Value
Number of lymph nodes removed, median (IQR)	18 (IQR, 13 to 24)	17 (IQR, 12 to 22)	0.454
N1 lymph nodes removed	6.5 (IQR, 4.5 to 9)	7 (IQR, 5 to 11)	0.323
N2 lymph nodes removed	11 (IQR, 7 to 15)	9 (IQR 6 to 13)	0.080
Patients with a minimal number of lymph nodes removed, *n* (%)			
Patients with ≥6 nodes removed	96 (100)	94 (100)	1.000
Patients with ≥18 nodes removed	49 (51.0)	44 (46.8)	0.559
Number of lymph nodes stations removed, median (IQR)	6 (IQR, 5 to 7)	6 (IQR, 5 to 7)	0.017 *
N1 stations	3 (IQR, 2 to 3)	3 (IQR, 2 to 3)	0.600
N2 stations	4 (IQR, 3 to 4)	3 (IQR, 3 to 4)	0.017 *
Adherence to the lymphadenectomy guidelines, *n* (%)			
National Comprehensive Cancer Network	87 (90.6)	98 (83.0)	0.119
IASLC L-SMLND for all stations	39 (40.6)	16 (17.0)	<0.001 *
IASLC L-SMLND for N2 stations	58 (60.4)	36 (38.3)	0.002 *
Clinical N stage, *n* (%)			0.276
cN0	87 (90.6)	82 (87.2)	
cN1	9 (9.4)	12 (12.8)	
Pathological N stage, *n* (%)			0.316
pN0	76 (79.2)	70 (74.5)	
pN1	13 (13.5)	11 (11.7)	
pN2	7 (7.3)	13 (13.8)	
Nodal upstaging cN0 to pN1 or pN2, *n* (%)	16 (18.4)	13 (15.8)	0.688

IASLC = International Association for the Study of Lung Cancer; IQR = interquartile range; L-SMLND = lobe-specific mediastinal lymph node dissection. * Statistically significant (*p* < 0.05).

**Table 5:** Univariable analysis of variables associated with compliance to the lobe-specific mediastinal lymphadenectomy guidelines.

Variables	Did Surgery Met the Lobe-Specific MLND IASCL Guidelines for N2 Lymph Nodes?		*p* Value
	YES	NO	
Age, years, mean (SD)	65.8 (SD: 7.7)	67.0 (SD: 6.6)	0.240
Sex, *n* (%)			0.045 *
Male	46 (41.8)	64 (58.2)	
Female	48 (60.0)	32 (40.0)	
Body Mass Index, kg/m^2^, mean (SD)	26.3 (SD: 4.3)	27.1 (SD: 5.2)	0.277
ppFEV1%, mean (SD)	64.5 (SD: 17.0)	62.8 (SD: 16.1)	0.395
Thoracic Revised Cardiac Risk Index, *n* (%)			0.041 *
Class A	81 (53.3)	71 (46.7)	
Class B	13 (37.1)	22 (62.9)	
Class C	0 (0)	3 (100.0)	
Charlson Comorbidity Index, median (IQR)	5 (IQR, 2 to 8)	6 (IQR, 3 to 9)	0.009 *
Histology, *n* (%)			0.249
Adenocarcinoma	51 (49.0)	53 (51.0)	
Squamous cell carcinoma	34 (55.7)	27 (44.3)	
Other	9 (36.0)	16 (64.0)	
Clinical T stage, *n* (%)			0.052
cT1	55 (56.7)	42 (43.3)	
cT2	32 (42.1)	44 (57.9)	
cT3	4 (28.6)	10 (71.4)	
cT4	3 (100)	0 (0)	
Clinical N stage, *n* (%)			0.456
cN0	82 (48.5)	87 (51.5)	
cN1	12 (57.1)	9 (42.9)	
Side of operation, *n* (%)			0.868
Right	54 (50.0)	54 (50.0)	
Left	40 (48.8)	42 (51.2)	
Extent of surgery, *n* (%)			0.018 *
Right upper lobectomy	39 (55.7)	31 (44.3)	
Right middle lobectomy	3 (25.0)	9 (75.0)	
Right lower lobectomy	12 (46.2)	14 (53.8)	
Left upper lobectomy	31 (60.8)	20 (39.2)	
Left lower lobectomy	9 (29.0)	22 (71.0)	
Type of electrosurgical device, *n* (%)			0.002 *
LigaSure device	58 (60.4)	38 (39.6)	
Monopolar device	36 (38.3)	58 (61.7)	
Type of thoracoscopic approach, *n* (%)			0.551
Multiportal	86 (48.9)	90 (51.1)	
Uniportal	8 (57.1)	6 (42.9)	

ppFEV_1_% = predicted postoperative percentage of calculated forced expiratory volume in 1 s; IQR = interquartile range. * Statistically significant (*p* < 0.05).

## Data Availability

The data underlying this article will be shared on reasonable request to the corresponding author.

## Appendix A

The IASLC definition of lobe-specific systematic nodal dissection [3,5]:

1. For right upper and middle lobes: subcarinal, superior and inferior paratracheal lymph nodes.
2. For right lower lobe: subcarinal, right inferior paratracheal and the paraesophageal or pulmonary ligament lymph nodes.
3. For left upper lobe: subcarinal, subaortic and para-aortic lymph nodes.
4. For left lower lobe: subcarinal, paraesophageal and pulmonary ligament lymph nodes.
5. For all lobes: dissection and histological examination of hilar and intrapulmonary (lobar, interlobar, segmental) lymph nodes.

The IASLC definition of complete resection—must fulfil all of the following [3,5]:

1. The resection margins (bronchial, vascular, peribronchial, around the tumor or the margins of any resected tissue) must be free of tumor proved microscopically.
2. The lung resection has to be accompanied by a systematic nodal dissection or by a lobe-specific systematic nodal dissection. The minimum number of removed lymph nodes was considered to be at least six: three from the intrapulmonary and/or hilar nodal stations and three from the mediastinal nodal stations, always including the subcarinal.
3. The capsule of those nodes removed separately and those located at the margin of the main lung specimen must be intact without extracapsular tumor invasion.
4. The highest mediastinal lymph node removed must be free of tumor.
